# Supplementary material for: Association of ideal cardiovascular health metrics with incident low estimated glomerular filtration rate: More than a decade follow-up in the Tehran Lipid and Glucose Study (TLGS)
Source: PLoS One. 2024 Feb 1;19(2):e0282773. doi: 10.1371/journal.pone.0282773 (PMC10833558; doi:10.1371/journal.pone.0282773)
Supplement: S1 File — (DOCX) [file pone.0282773.s001.docx]

| Table S1. Proportional hazards assumption in the Cox model of intermediate and ideal categories of each ICVHM† for incident low eGFR | | | | |
| --- | --- | --- | --- | --- |
|  |  | Model 1 | Model 2 | Model 3 |
|  | e/N | P value | P value | P value |
| Smoking status |  |  |  |  |
| Poor | 113/838 | 1 | 1 | 1 |
| Intermediate | 89/510 | 0.63 | 0.62 | 0.88 |
| Ideal | 1057/5579 | 0.65 | 0.58 | 0.75 |
| Body mass index |  |  |  |  |
| Poor | 463/1732 | 1 | 1 | 1 |
| Intermediate | 559/2883 | 0.63 | 0.61 | 0.30 |
| Ideal | 237/2312 | 0.35 | 0.48 | 0.87 |
| Physical activity |  |  |  |  |
| Poor | 402/2567 | 1 | 1 | 1 |
| Intermediate | 297/1516 | 0.33 | 0.34 | 0.29 |
| Ideal | 560/2844 | 0.90 | 0.84 | 0.95 |
| Total cholesterol |  |  |  |  |
| Poor | 207/644 | 1 | 1 | 1 |
| Intermediate | 470/1778 | 0.18 | 0.17 | 0.30 |
| Ideal | 582/4505 | 0.80 | 0.80 | 0.82 |
| Blood pressure |  |  |  |  |
| Poor | 236/620 | 1 | 1 | 1 |
| Intermediate | 455/1975 | 0.83 | 0.90 | 0.90 |
| Ideal | 568/4332 | 0.38 | 0.33 | 0.35 |
| Fasting blood glucose |  |  |  |  |
| Poor | 144/354 | 1 | 1 | 1 |
| Intermediate | 232/761 | 0.54 | 0.56 | 0.73 |
| Ideal | 883/5812 | 0.91 | 0.98 | 0.85 |
| ICVHM: ideal cardiovascular health metrics; eGFR: estimated glomerular filtration rate; e/N, number of low eGFR events /number of population at risk.  † Defined according to the number of ideal metrics: 0 to 1 (poor), 2 to 4 (intermediate), and 5 to 6 (ideal).  Model 1: adjusted for gender and age; Model 2: further adjusted for educational level and marital status; Model 3: further adjusted for eGFR. | | | | |

| Table S2. Proportional hazards assumption in the Cox model of ICVHM† for incident low eGFR (per one additional metric) | | | |
| --- | --- | --- | --- |
|  | Model 1 | Model 2 | Model 3 |
|  | P value | P value | P value |
| Global cardiovascular health |  |  |  |
| Poor | 1 | 1 | 1 |
| Intermediate | 0.89 | 0.94 | 0.79 |
| Ideal | 0.89 | 0.79 | 0.82 |
| Behavioral cardiovascular health | 0.19 | 0.21 | 0.38 |
| Biological cardiovascular health | 0.12 | 0.10 | 0.12 |
| ICVH: ideal cardiovascular health; eGFR: estimated glomerular filtration rate.  † Defined according to the number of ideal metrics: 0 to 1 (poor), 2 to 4 (intermediate), and 5 to 6 (ideal).  Model 1: adjusted for gender and age; Model 2: further adjusted for educational level and marital status; Model 3: further adjusted for eGFR. | | | |

| Table S3. HRs and 95% CIs of intermediate and ideal categories of each ICVHM for incident low eGFR, further adjusted for propensity score | | | | |
| --- | --- | --- | --- | --- |
|  |  | Model 1 | Model 2 | Model 3 |
|  | e/N | HR (95% CI) | HR (95% CI) | HR (95% CI) |
| Smoking status |  |  |  |  |
| Poor | 113/838 | 1 | 1 | 1 |
| Intermediate | 89/510 | 0.77 (0.57-1.03) | **0.70 (0.52-0.94)** | 0.91 (0.68-1.22) |
| Ideal | 1057/5579 | 0.91 (0.74-1.13) | 0.91 (0.74-1.12) | 0.95 (0.77-1.17) |
| Body mass index |  |  |  |  |
| Poor | 463/1732 | 1 | 1 | 1 |
| Intermediate | 559/2883 | 0.91 (0.81-1.04) | 0.91 (0.80-1.03) | **0.87 (0.76-0.98)** |
| Ideal | 237/2312 | **0.86 (0.73-1.01)** * | **0.85 (0.72-1.00)** * | **0.83 (0.71-0.98)** |
| Physical activity |  |  |  |  |
| Poor | 402/2567 | 1 | 1 | 1 |
| Intermediate | 297/1516 | 1.02 (0.87-1.18) | 1.00 (0.86-1.17) | 1.03 (0.88-1.19) |
| Ideal | 560/2844 | 1.04 (0.91-1.18) | 1.04 (0.92-1.18) | 1.04 (0.91-1.18) |
| Total cholesterol |  |  |  |  |
| Poor | 207/644 | 1 | 1 | 1 |
| Intermediate | 470/1778 | 0.95 (0.80-1.11) | 0.90 (0.76-1.07) | 0.99 (0.84-1.18) |
| Ideal | 582/4505 | **0.81 (0.69-0.96)** | **0.77 (0.65-0.91)** | 0.89 (0.75-1.06) |
| Blood pressure |  |  |  |  |
| Poor | 236/620 | 1 | 1 | 1 |
| Intermediate | 455/1975 | **0.79 (0.67-0.92)** | **0.78 (0.67-0.92)** | **0.80 (0.68-0.93)** |
| Ideal | 568/4332 | **0.71 (0.61-0.84)** | **0.70 (0.60-0.82)** | **0.71 (0.60-0.83)** |
| Fasting blood glucose |  |  |  |  |
| Poor | 144/354 | 1 | 1 | 1 |
| Intermediate | 232/761 | 0.93 (0.75-1.15) | 0.89 (0.72-1.10) | 0.85 (0.68-1.05) |
| Ideal | 883/5812 | **0.80 (0.66-0.96)** | **0.76 (0.63-0.91)** | **0.77 (0.64-0.93)** |
| HR, hazard ratio; CI, confidence interval; ICVHM: ideal cardiovascular health metrics; eGFR: estimated glomerular filtration rate; e/N, number of low eGFR events /number of population at risk.  Model 1: adjusted for gender and age; Model 2: further adjusted for educational level and marital status; Model 3: further adjusted for eGFR.  Significant values are bold (P value < 0.05)  *P value = 0.06 | | | | |

| Table S4. Cox proportional hazard models of ICVH for incident low eGFR (per one additional metric), further adjusted for propensity score | | | |
| --- | --- | --- | --- |
|  | Model 1 | Model 2 | Model 3 |
|  | HR (95% CI) | HR (95% CI) | HR (95% CI) |
| Global cardiovascular health | **0.92 (0.88-0.97)** | **0.92 (0.87-0.96)** | **0.93(0.88-0.97)** |
| Behavioral cardiovascular health | 0.99 (0.92-1.07) | 1.00 (0.93-1.08) | 0.98(0.91-1.06) |
| Biological cardiovascular health | **0.87 (0.81-0.92)** | **0.85 (0.80-0.90)** | **0.88 (0.82-0.94)** |
| ICVH: ideal cardiovascular health; eGFR: estimated glomerular filtration rate; HR, hazard ratio; CI, confidence interval.  Model 1: adjusted for gender and age; Model 2: further adjusted for educational level and marital status; Model 3: further adjusted for eGFR. | | | |

| Table S5. HRs and 95% CIs of different global cardiovascular health status † for incident low eGFR, further adjusted for propensity score | | | | |
| --- | --- | --- | --- | --- |
|  | | Model 1 | Model 2 | Model 3 |
|  | | HR (95% CI) | HR (95% CI) | HR (95% CI) |
| Global Cardiovascular health | | | | |
|  | Poor | 1 | 1 | 1 |
|  | Intermediate | **0.89 (0.78-1.00) *** | **0.87 (0.77-0.98)** | **0.89 (0.79-1.01)*** |
|  | Ideal | **0.71 (0.59-0.86)** | **0.69 (0.57-0.84)** | **0.72 (0.59-0.87)** |
| Age, year | | 1.11 (1.10-1.11) | 1.11 (1.10-1.12) | 1.08 (1.07-1.09) |
| Female (male as reference) | | 2.00 (1.76-2.25) | 2.20 (1.93-2.51) | 1.54 (1.34-1.76) |
| Educational level | | | | |
|  | > 12 |  | 1 | 1 |
|  | 6-12 | ـ | 1.15 (0.96-1.38) | 1.15 (0.96-1.38) |
|  | < 6 | ـ | **0.79 (0.64-0.96)** | 0.96 (0.78-1.17) |
| Marital status | | | | |
|  | Married |  | 1 | 1 |
|  | Divorced + Widowed |  | 0.97 (0.79-1.19) | 0.91 (0.74-1.12) |
|  | Single |  | 1.05 (0.75-1.47) | 0.92 (0.66-1.29) |
| eGFR | |  | ـ | **0.93 (0.93-0.94)** |
| HR, Hazard Ratio; CI, confidence interval; eGFR, estimated glomerular filtration rate.  † Defined according to the number of ideal metrics: 0 to 1 (poor), 2 to 4 (intermediate), and 5 to 6 (ideal).  Model 1: adjusted for gender and age; Model 2: further adjusted for educational level marital status; Model 3: further adjusted for estimated glomerular filtration rate.  * P = 0.06 | | | | |

| Table S6. HRs and 95% CIs of intermediate and ideal categories of each ICVHM for incident low eGFR, using interval-censoring approach | | | | |
| --- | --- | --- | --- | --- |
|  |  | Model 1 | Model 2 | Model 3 |
|  | e/N | HR (95% CI) | HR (95% CI) | HR (95% CI) |
| Smoking status |  | | | |
| Poor | 113/838 | 1 | 1 | 1 |
| Intermediate | 89/510 | 0.88 (0.64-1.21) | 0.88 (0.65-1.21) | 0.88 (0.63-1.21) |
| Ideal | 1057/5579 | 0.96 (0.75-1.21) | 0.97 (0.75-1.24) | 0.95 (0.76-1.20) |
| Body mass index |  | | | |
| Poor | 463/1732 | 1 | 1 | 1 |
| Intermediate | 559/2883 | 0.91 (0.80-1.03) | 0.89 (0.78-1.02) | **0.87 (0.77-0.99)** |
| Ideal | 237/2312 | **0.85 (0.73-0.98)** | **0.83 (0.71-0.97)** | **0.84 (0.72-0.98)** |
| Physical activity |  | | | |
| Poor | 402/2567 | 1 | 1 | 1 |
| Intermediate | 297/1516 | 1.01 (0.91-1.13) | 1.00 (0.85-1.18) | 1.03 (0.87-1.21) |
| Ideal | 560/2844 | 1.04 (0.91-1.18) | 1.03 (0.90-1.17) | 1.05 (0.92-1.19) |
| Total cholesterol |  | | | |
| Poor | 207/644 | 1 | 1 | 1 |
| Intermediate | 470/1778 | 1.00 (0.85-1.17) | 0.99 (0.83-1.18) | 0.99 (0.83-1.17) |
| Ideal | 582/4505 | 0.87 (0.74-1.01) | 0.85 (0.72-1.02) | 0.88 (0.75-1.04) |
| Blood pressure | | | | |
| Poor | 236/620 | 1 | 1 | 1 |
| Intermediate | 455/1975 | **0.79 (0.66-0.95)** | **0.79 (0.66-0.94)** | **0.79 (0.68-0.91)** |
| Ideal | 568/4332 | **0.72 (0.59-0.87)** | **0.71 (0.60-0.84)** | **0.70 (0.58-0.84)** |
| Fasting blood glucose | | | | |
| Poor | 144/354 | 1 | 1 | 1 |
| Intermediate | 232/761 | 0.97 (0.80-1.17) | 0.95 (0.78-1.16) | 0.84 (0.68-1.03) |
| Ideal | 883/5812 | 0.83 (0.68-1.02) | **0.81 (0.68-0.97)** | **0.75 (0.62-0.92)** |
| HR, hazard ratio; CI, confidence interval; ICVHM: ideal cardiovascular health metrics; eGFR: estimated glomerular filtration rate; e/N, number of low eGFR events /number of population at risk.  Model 1: adjusted for gender and age; Model 2: further adjusted for educational level and marital status; Model 3: further adjusted for eGFR.  Significant values are bold (P value < 0.05) | | | | |

| Table S7. Cox proportional hazard models of ICVH for incident low eGFR (per one additional metric), using interval-censoring approach | | | |
| --- | --- | --- | --- |
|  | Model 1 | Model 2 | Model 3 |
|  | HR (95% CI) | HR (95% CI) | HR (95% CI) |
| Global cardiovascular health | **0.92 (0.89-0.96)** | **0.92 (0.88-0.96)** | **0.92 (0.88-0.97)** |
| Behavioral cardiovascular health | 0.99 (0.92-1.06) | 0.99 (0.91-1.06) | 0.99 (0.91-1.08) |
| Biological cardiovascular health | **0.88 (0.82-0.94)** | **0.87 (0.82-0.92)** | **0.87 (0.82-0.93)** |
| ICVH: ideal cardiovascular health; eGFR: estimated glomerular filtration rate; HR, hazard ratio; CI, confidence interval.  Model 1: adjusted for gender and age; Model 2: further adjusted for educational level and marital status; Model 3: further adjusted for eGFR. | | | |

| Table S8. HRs and 95% CIs of different global cardiovascular health status † for incident low eGFR, using interval-censoring approach | | | | |
| --- | --- | --- | --- | --- |
|  | | Model 1 | Model 2 | Model 3 |
|  | | HR (95% CI) | HR (95% CI) | HR (95% CI) |
| Global Cardiovascular health | | | | |
|  | Poor | 1 | 1 | 1 |
|  | Intermediate | **0.89 (0.80-0.99)** | **0.88 (0.79-0.98)** | **0.88 (0.78-0.99)** |
|  | Ideal | **0.72 (0.56-0.91)** | **0.70 (0.56-0.88)** | **0.71 (0.59-0.86)** |
| Age, year | | 1.11 (1.10-1.12) | 1.12 (1.11-1.13) | 1.09 (1.08-1.09) |
| Female (male as reference) | | 1.94 (1.70-2.20) | 2.10 (1.79-2.42) | 1.56 (1.40-1.75) |
| Educational level | | | | |
|  | > 12 | 1 | 1 | 1 |
|  | 6-12 |  | 1.05 (0.88-1.25) | 1.16 (0.94-1.43) |
|  | < 6 |  | **0.80 (0.65-0.99)** | 0.95 (0.79-1.16) |
| Marital status | | | | |
|  | Married | 1 | 1 | 1 |
|  | Divorced + Widowed |  | 0.88 (0.70-1.11) | 0.94 (0.77-1.15) |
|  | Single |  | 0.94 (0.66-1.34) | 1.00 (0.75-1.33) |
| eGFR | |  |  | **0.93 (0.93-0.94)** |
| HR, Hazard Ratio; CI, confidence interval; eGFR, estimated glomerular filtration rate.  † Defined according to the number of ideal metrics: 0 to 1 (poor), 2 to 4 (intermediate), and 5 to 6 (ideal).  Model 1: adjusted for gender and age; Model 2: further adjusted for educational level marital status; Model 3: further adjusted for estimated glomerular filtration rate. | | | | |

| Table S9. HRs and 95% CIs of intermediate and ideal categories of each ICVHM for incident low eGFR, using age and marital status as time-varying analysis | | | | |
| --- | --- | --- | --- | --- |
|  |  | Model 1 | Model 2 | Model 3 |
|  | e/N | HR (95% CI) | HR (95% CI) | HR (95% CI) |
| Smoking status |  |  | | |
| Poor | 113/838 | 1 | 1 | 1 |
| Intermediate | 89/510 | 0.88 (0.67-1.16) | 0.88 (0.67-1.17) | 0.86 (0.65-1.13) |
| Ideal | 1057/5579 | 0.94 (0.76-1.15) | 0.95 (0.77-1.17) | 0.94 (0.76-1.16) |
| Body mass index |  |  | | |
| Poor | 463/1732 | 1 | 1 | 1 |
| Intermediate | 559/2883 | **0.92 (0.81-1.00)** * | 0.90 (0.79-1.02) | **0.88 (0.77-0.99)** |
| Ideal | 237/2312 | **0.86 (0.73-1.01)** * | **0.83 (0.70-0.98)** | **0.84 (0.71-0.99)** |
| Physical activity |  |  | | |
| Poor | 402/2567 | 1 | 1 | 1 |
| Intermediate | 297/1516 | 1.02 (0.87-1.18) | 1.01 (0.87-1.18) | 1.03 (0.89-1.19) |
| Ideal | 560/2844 | 1.04 (0.91-1.18) | 1.04 (0.91-1.18) | 1.04 (0.92-1.19) |
| Total cholesterol |  |  | | |
| Poor | 207/644 | 1 | 1 | 1 |
| Intermediate | 470/1778 | 0.99 (0.84-1.17) | 0.98 (0.83-1.16) | 0.99 (0.84-1.18) |
| Ideal | 582/4505 | 0.89 (0.75-1.05) | 0.87 (0.74-1.03) | 0.94 (0.80-1.12) |
| Blood pressure |  |  | | |
| Poor | 236/620 | 1 | 1 | 1 |
| Intermediate | 455/1975 | **0.80 (0.68-0.95)** | **0.80 (0.68-0.94)** | **0.83 (0.70-0.97)** |
| Ideal | 568/4332 | **0.75 (0.64-0.86)** | **0.73 (0.63-0.86)** | **0.78 (0.67-0.92)** |
| Fasting blood glucose |  |  | | |
| Poor | 144/354 | 1 | 1 | 1 |
| Intermediate | 232/761 | 0.96 (0.78-1.19) | 0.95 (0.77-1.17) | 0.85 (0.69-1.06) |
| Ideal | 883/5812 | **0.84 (0.70-1.01)** * | **0.82 (0.68-0.98)** | **0.80 (0.66-0.96)** |
| HR, hazard ratio; CI, confidence interval; ICVHM: ideal cardiovascular health metrics; eGFR: estimated glomerular filtration rate; e/N, number of low eGFR events /number of population at risk.  Model 1: adjusted for gender and age; Model 2: further adjusted for educational level and marital status; Model 3: further adjusted for eGFR.  Significant values are bold (P value < 0.05)  *P value = 0.06 | | | | |

| Table S10. Cox proportional hazard models of ICVH for incident low eGFR (per one additional metric), using age and marital status as time-varying analysis | | | |
| --- | --- | --- | --- |
|  | Model 1 | Model 2 | Model 3 |
|  | HR (95% CI) | HR (95% CI) | HR (95% CI) |
| Global cardiovascular health | **0.94 (0.89-0.98)** | **0.93 (0.89-0.97)** | **0.94 (0.90-0.98)** |
| Behavioral cardiovascular health | 0.98 (0.91-1.06) | 0.98 (0.91-1.06) | 1.00 (0.93-1.07) |
| Biological cardiovascular health | **0.90 (0.84-0.96)** | **0.89 (0.83-0.95)** | **0.90 (0.85-0.96)** |
| ICVH: ideal cardiovascular health; eGFR: estimated glomerular filtration rate; HR, hazard ratio; CI, confidence interval.  Model 1: adjusted for gender and age; Model 2: further adjusted for educational level and marital status; Model 3: further adjusted for eGFR. | | | |

| Table S11. HRs and 95% CIs of different global cardiovascular health status † for incident low eGFR, using age and marital status as time-varying analysis | | | | |
| --- | --- | --- | --- | --- |
|  | | Model 1 | Model 2 | Model 3 |
|  | | HR (95% CI) | HR (95% CI) | HR (95% CI) |
| Global Cardiovascular health | | | | |
|  | Poor | 1 | 1 | 1 |
|  | Intermediate | 0.91 (0.81-1.04) | 0.90 (0.79-1.02) | 0.93 (0.82-1.05) |
|  | Ideal | **0.75 (0.62-0.92)** | **0.72 (0.59-0.88)** | **0.79 (0.65-0.96)** |
| Age, year | | 1.11 (1.11-1.12) | 1.12 (1.11-1.13) | 1.10 (1.09-1.10) |
| Female (male as reference) | | 1.90 (1.68-2.14) | 2.03 (1.79-2.23) | 1.58 (1.39-1.81) |
| Educational level | | | | |
|  | > 12 | 1 | 1 | 1 |
|  | 6-12 |  | 1.07 (0.90-1.28) | 1.20 (1.01-1.42) |
|  | < 6 |  | **0.79 (0.65-0.97)** | 1.00 (0.83-1.22) |
| Marital status | | | | |
|  | Married | 1 | 1 | 1 |
|  | Divorced + Widowed |  | 0.95 (0.79-1.14) | 0.94 (0.79-1.13) |
|  | Single |  | 1.17 (0.85-1.62) | 1.17 (0.85-1.61) |
| eGFR | |  |  | **0.90 (0.89-0.91)** |
| HR, Hazard Ratio; CI, confidence interval; eGFR, estimated glomerular filtration rate.  † Defined according to the number of ideal metrics: 0 to 1 (poor), 2 to 4 (intermediate), and 5 to 6 (ideal).  Model 1: adjusted for gender and age; Model 2: further adjusted for educational level marital status; Model 3: further adjusted for estimated glomerular filtration rate. | | | | |

| Table S12. Comparison of baseline characteristics between respondent and non-respondent groups | | | | | |
| --- | --- | --- | --- | --- | --- |
| Variables | | | **Respondent**  (n = 6927) | **Non-Respondent**  (n = 939) | Mean difference (CI) |
| Continuous variables | | |  | | |
|  | Age (year) | | 38.73 | 36.32 | 2.41 (1.56-3.25) |
|  | BMI (kg/m^2^) | | 27.23 | 26.58 | 0.65 (0.22-1.10) |
|  | SBP (mmHg) | | 111.21 | 109.79 | 1.42 (0.22-2.63) |
|  | DBP (mmHg) | | 72.96 | 71.75 | 1.21 (0.41-1.99) |
|  | FPG (mmol/L) | | 5.16 | 5.22 | -0.06 (-0.19-0.06) |
|  | TC (mmol/L) | | 4.829 | 4.826 | 0.003 (-0.072-0.076) |
|  | eGFR (ml/min/1.73 m^2^) | | 85.01 | 87.58 | -2.57 (-3.65- -1.48) |
| Categorical variables | | |  | | |
|  | Gender (male) (%) | | 42.47 | 39.72 | 2.75 (0.59-5.57) |
|  | Educational level (year) (%) | |  | | |
|  | | < 6 | 16.44 | 13.36 | 3.08 (0.59-5.57) |
|  | | 6-12 | 61.07 | 62.62 | -1.55 (-5.07-1.95) |
|  | | > 12 | 22.49 | 24.02 | -1.53 (-4.62-1.56) |
|  | Physical activity (METS) (%) | |  | | |
|  | | < 600 | 37.06 | 39.29 | -2.23 (-6.17-1.71) |
|  | | 600-1500 | 21.89 | 20.81 | 1.08 (-2.20-2.39) |
|  | | > 1500 | 41.06 | 39.91 | 1.15 (-2.80-5.11) |
|  | Smoking (%) | |  | | |
|  | | Current | 12.10 | 17.13 | -5.03 (-7.91-2.16) |
|  | | Past | 7.40 | 5.62 | 1.78 (-0.05-3.54) |
|  | | Never | 80.54 | 77.25 | 3.29 (0.07-6.51) |
|  | Marital status (%) | |  | | |
|  | | Married | 77.72 | 70.05 | 7.67 (4.58-10.77) |
|  | | Widowed + Divorced | 3.82 | 3.96 | -0.14 (-1.46-1.19) |
|  | | Single | 18.45 | 25.99 | -7.54 (-10.49-4.58) |
|  | Glucose lowering drug use, yes (%) | | 3.46 | 2.45 | 1.01 (-0.06-2.09) |
|  | Anti-hypertensive drug use, yes (%) | | 2.34 | 2.13 | 0.21 (-0.78-1.19) |
|  | Lipid-lowering drug use, yes (%) | | 2.78 | 1.49% | 1.29 (0.42-2.16) |
| Values are mean, median(only for FPG), or frequency.  BMI, body mass index; SBP, systolic blood pressure; DBP, diastolic blood pressure; FPG, fasting plasma glucose; TC, total cholesterol; eGFR, estimated glomerular filtration rate; METS, metabolic equivalent of task. | | | | | |

| Table S13. HRs and 95% CIs of intermediate/ideal compared to poor categories of nutritional status for incident low eGFR in a subgroup with nutritional data (n = 2285) | | | | |
| --- | --- | --- | --- | --- |
| Healthy diet | e/N | Model 1 | Model 2 | Model 3 |
|  |  | HR (95% CI) | HR (95% CI) | HR (95% CI) |
| Poor | 184 / 1343 | 1 | 1 | 1 |
| Intermediate/Ideal | 192 / 942 | 0.96 (0.88-1.05) | 0.97 (0.88-1.05) | 0.96 (0.88-1.04) |
| HR, hazard ratio; CI, confidence interval; eGFR, estimated glomerular filtration rate; e/N: number of low eGFR events /number of population at risk.  Model 1: adjusted for gender and age. Model 2: further adjusted for educational level and marital status Model 3: further adjusted for eGFR | | | | |

| **Table S14**. HRs and 95% CIs for the incident low eGFR per one additional ICVHM in a subgroup with dietary data (n = 2285) | | | |
| --- | --- | --- | --- |
| **Cardiovascular Health** | HR (95 % CI) | | |
| **Global cardiovascular health** | 0.98 (0.95-1.01) | 0.98 (0.94-1.01) | 0.98 (0.94-1.01) |
| **Behavioral cardiovascular health** | 0.99 (0.94-1.04) | 0.98 (0.93-1.03) | 0.98 (0.93-1.02) |
| **Biological cardiovascular health** | 0.97 (0.92-1.02) | 0.97 (0.92-1.02) | 0.97 (0.92-1.02) |
| HR, hazard ratio; CI, confidence interval; eGFR, estimated glomerular filtration rate; ICVHM: ideal cardiovascular health metrics.  Model 1: adjusted for gender and age. Model 2: further adjusted for educational level, marital status. Model 3: further adjusted for eGFR. | | | |

| Table S15. HRs and 95% CIs of the association between different categories of nutritional status with incident low eGFR in a subgroup with dietary data (n = 2285) | | | | |
| --- | --- | --- | --- | --- |
| Cardiovascular health* | | Model 1 | Model 2 | Model 3 |
|  | Poor | 1 | 1 | 1 |
|  | Intermediate | 0.93 (0.81-1.06) | 0.92 (0.81-1.05) | 0.92 (0.81-1.05) |
|  | Ideal | 0.94 (0.82-1.09) | 0.93 (0.81-1.06) | 0.93 (0.80-1.06) |
| HRs, hazard ratio; CI, confidence interval; eGFR, estimated glomerular filtration rate  *Defined according to the number of ideal metrics: 0 to 1 (poor), 2 to 4 (intermediate) and 5 to 7 (ideal). Model 1: adjusted for gender and age. Model 2: further adjusted for educational level, marital status. Model 3: further adjusted for eGFR. | | | | |
