# Supplementary material for: Association of ideal cardiovascular health metrics with incident low estimated glomerular filtration rate: More than a decade follow-up in the Tehran Lipid and Glucose Study (TLGS)
Source: PLoS One. 2024 Feb 1;19(2):e0282773. doi: 10.1371/journal.pone.0282773 (PMC10833558; doi:10.1371/journal.pone.0282773)
Supplement: Attachment — Submitted filename: Response to reviewers.docx [file pone.0282773.s003.docx]

Top of Form

**View Letter**

Bottom of Form

| Date: | Jul 18 2022 02:30PM |
| --- | --- |
| To: | "maryam tohidi" tohidi@endocrine.ac.ir;farzadhadaegh@gmail.com |
| From: | "PLOS ONE" plosone@plos.org |
| Subject: | PLOS ONE Decision: Revision required [PONE-D-22-17476] |

PONE-D-22-17476

Association of Ideal Cardiovascular Health Metrics with Incident Chronic Kidney Disease: more than a decade follow-up in the Tehran Lipid and Glucose Study (TLGS)

PLOS ONE

Journal Requirements:

When submitting your revision, we need you to address these additional requirements.

1. Please ensure that your manuscript meets PLOS ONE's style requirements, including those for file naming. The PLOS ONE style templates can be found at 
https://journals.plos.org/plosone/s/file?id=wjVg/PLOSOne_formatting_sample_main_body.pdf and 
https://journals.plos.org/plosone/s/file?id=ba62/PLOSOne_formatting_sample_title_authors_affiliations.pdf

**Response:** Thank you, done.

2. We note that your study is based on the clinical trials described in the references 20 and 21. Please provide the clinical trial registration numbers for these trials in your manuscript.

### Response: Agreed, done, the trial registration number (ISRCTN52588395) was added to the text. ” (page, line: 89)

3. We note that the grant information you provided in the ‘Funding Information’ and ‘Financial Disclosure’ sections do not match.

When you resubmit, please ensure that you provide the correct grant numbers for the awards you received for your study in the ‘Funding Information’ section.

**Response:** Agreed, corrected. "This study was initially supported in part by grant No. 121 from the National Research Council of the Islamic Republic of Iran and then by grant No 30382 from the Shahid Beheshti University of Medical Sciences." ” (page, line: 100-103)

4. Please state what role the funders took in the study.  If the funders had no role, please state: "The funders had no role in study design, data collection and analysis, decision to publish, or preparation of the manuscript." 
If this statement is not correct you must amend it as needed. 
Please include this amended Role of Funder statement in your cover letter; we will change the online submission form on your behalf.

**Response:** The authors declare that they have no competing interests.

5. Your ethics statement should only appear in the Methods section of your manuscript. If your ethics statement is written in any section besides the Methods, please move it to the Methods section and delete it from any other section. Please ensure that your ethics statement is included in your manuscript, as the ethics statement entered into the online submission form will not be published alongside your manuscript.

**Response:** Agreed, the ethic statement and related approval number was mentioned in the Method section. ” (page, line: 100-103)

[Note: HTML markup is below. Please do not edit.]

Reviewers' comments:

Reviewer's Responses to Questions

**Comments to the Author**

1. Is the manuscript technically sound, and do the data support the conclusions?

The manuscript must describe a technically sound piece of scientific research with data that supports the conclusions. Experiments must have been conducted rigorously, with appropriate controls, replication, and sample sizes. The conclusions must be drawn appropriately based on the data presented.

Reviewer #1: Yes

Reviewer #2: Partly

2. Has the statistical analysis been performed appropriately and rigorously?

Reviewer #1: Yes

Reviewer #2: I Don't Know

3. Have the authors made all data underlying the findings in their manuscript fully available?

The [PLOS Data policy](http://www.plosone.org/static/policies.action#sharing) requires authors to make all data underlying the findings described in their manuscript fully available without restriction, with rare exception (please refer to the Data Availability Statement in the manuscript PDF file). The data should be provided as part of the manuscript or its supporting information, or deposited to a public repository. For example, in addition to summary statistics, the data points behind means, medians and variance measures should be available. If there are restrictions on publicly sharing data—e.g. participant privacy or use of data from a third party—those must be specified.

Reviewer #1: Yes

Reviewer #2: Yes

4. Is the manuscript presented in an intelligible fashion and written in standard English?

PLOS ONE does not copyedit accepted manuscripts, so the language in submitted articles must be clear, correct, and unambiguous. Any typographical or grammatical errors should be corrected at revision, so please note any specific errors here.

Reviewer #1: Yes

Reviewer #2: Yes

5. Review Comments to the Author

Please use the space provided to explain your answers to the questions above. You may also include additional comments for the author, including concerns about dual publication, research ethics, or publication ethics. (Please upload your review as an attachment if it exceeds 20,000 characters)

Reviewer #1: Alizadeh and colleagues carried out a prospective study examining the association between ideal cardiovascular health metrics and incident chronic kidney disease. In a cohort of 7388 patients, they found that ideal and intermediate categories of BMI, BP and ideal categories of FBP are associated with lower risk of incident CKD in middle-aged subjects while only intermediate and ideal categories of BP associated with lower CKD risk.

This manuscript could be of some interest, but I have several concerns. Briefly, my comments:
1) In the Abstract the following sentence needs to be rephrased because there are not only HR but also the percentage of risk reduction “the corresponding HRs (95% confidence intervals) were 13% (0.87, 0.77-0.99), 16% (0.84, 0.76-0.99), 21% (0.79, 0.68-0.93), 30% (0.70, 0.60-0.83) and 24% (0.76, 0.64-0.91)”. Furthermore, in the conclusion of abstract Authors stated that Results were found only in middle-aged while there are some findings also in elderly subjects. Abstract needs to be improved

**Response:** The mentioned part of the abstract was rephrased as below: “the corresponding HRs (95% confidence intervals) were (0.87, 0.77-0.99), (0.84, 0.76-0.99), (0.79, 0.68-0.93), (0.70, 0.60-0.83) and (0.76, 0.64-0.91).” (page , line: 37-39)

2) In Methods Section, Authors indicated that at baseline in TLGS were excluded subjects without CKD, but definition of this disease is not only based on GFR but also urinary anomalies such as proteinuria and albuminuria. Could Authors exclude that, at baseline, patients had a significant proteinuria? This is a crucial point in the evaluation of incident CKD. If data on proteinuria were not available, I suggest referring to low eGFR (<60 ml/min) as outcome instead of CKD.

**Response:** Thank you for this valuable comment. Since, in this cohort study, assay for urine protein was not performed at baseline and follow-up and CKD was defined based on eGFR, we corrected the manuscript and Tables according to this comment.” (page , line: The whole text)

3) Creatinine levels were assessed by kinetic colorimetric Jaffè and not with enzymatic assay, but CKD-EPI formula was used to estimate GFR. The Authors did not perform a correction on creatinine values to use CKD-EPI formula. This could limit GFR evaluation. I suggest that Since creatinine was not standardized to isotope dilution mass spectrometry values, Authors could reduce creatinine values by 5% as indicated by Skali et al. and, after, could use CKD-EPI formula (PMID: 21884875)

**Response:** Agreed, this issue for correcting the creatinine levels that has been considered in the data analysis, was added to the text. ” (page, line: 135-137)

4) Surprisingly, 68.3% of elderly subjects (>65 y) enrolled in the study developed incident CKD. I think that a selection bias could be the reason for this high incidence of CKD. Probably, Authors could limit the analyses on middle-aged population.

**Response:** Agreed, All information related to age over 65 years was removed from the draft article.

Reviewer #2: I’ve read with interest the draft “Association of ideal cardiovascular health metrics with incident chronic 2 kidney disease: more than a decade follow-up in the Tehran Lipid and 3 Glucose Study (TLGS)”, by Fatemeh Alizadeh, Maryam Tohidi, Mitra Hasheminia, Firoozeh Hosseini-Esfahani, Fereidoun Azizi, Farzad Hadaegh
However, some important issues have to be raised.

Introduction :
- Line 46-47. Authors should briefly introduce and define the main non-communicable diseases (NCDs).

**Response:** The mentioned items were corrected. ” (page, line: 46-49)

- Line 65-66. A multifactorial treatment aimed at multiple risk factors reduces the risk of cardiovascular events in populations with type 2 diabetes mellitus and renal impairment. This issue is addressed in some recent literature which should be added to the references. Gaede P et al, N Engl J Med. 2003;348(5):383-393. doi:10.1056/NEJMoa021778; Sasso FC et al Cardiovasc Diabetol. 2021;20(1):145. Published 2021 Jul 16. doi:10.1186/s12933-021-01343-1

**Response:** Thank you, for suggesting these references. The suggested references were added to the text. ” (page, line: 66-69)

Methods and Materials:
- Line 128-129. Font typing error.

**Response:** Thank you, the mentioned items were corrected. ” (page, line: 134-135)

Discussion:
- Line 251. “china” is a typing error.

**Response:** Thank you, the mentioned items were corrected. ” (page, line: 238)

- Line 237-239. “Among middle-aged participants, the intermediate and ideal categories of BMI and BP, and the ideal category of FPG and in elderly individuals, only intermediate and ideal BP was associated with reduced risk of CKD”. The authors should better clarify the pathophysiological mechanisms/hypotheses supporting this sentence.

**Response:** Thank you, all information related to age over 65 years was removed from the draft article according to the opinions of reviewers.

- Line 262. “Actually, no association was demonstrated between ICVHM and incident CKD among elder population”. It therefore seems that ICVHM does not affect the prognosis of elderly patients. However, some recent literature seems in contrast with this conclusion. The authors should better clarify the pathophysiological mechanisms/hypotheses supporting this sentence.

**Response:** agreed, according to comment number 4 of the first reviewer and the last comment of the second reviewer, we decided to remove participant over 65 years old from the draft of the article.

6. PLOS authors have the option to publish the peer review history of their article ([what does this mean?](https://journals.plos.org/plosone/s/editorial-and-peer-review-process#loc-peer-review-history)). If published, this will include your full peer review and any attached files.


If you choose “no”, your identity will remain anonymous but your review may still be made public.


**Do you want your identity to be public for this peer review?** For information about this choice, including consent withdrawal, please see our [Privacy Policy](https://www.plos.org/privacy-policy).

Reviewer #1: No

Reviewer #2: No
