# Supplementary material for: Association of ideal cardiovascular health metrics with incident low estimated glomerular filtration rate: More than a decade follow-up in the Tehran Lipid and Glucose Study (TLGS)
Source: PLoS One. 2024 Feb 1;19(2):e0282773. doi: 10.1371/journal.pone.0282773 (PMC10833558; doi:10.1371/journal.pone.0282773)
Supplement: Attachment — Submitted filename: Response to reviewers.docx [file pone.0282773.s004.docx]

Reviewer #1: All the criticisms raised were solved by the Authors. I do not have any further comment on the manuscript.

**Response: Thank you for the positive comment.**

Reviewer #2:

Dear Editor,
I’ve re revised the paper “Association of ideal cardiovascular health metrics with incident low estimated
glomerular filtration rate: more than a decade follow-up in the Tehran Lipid and
Glucose Study (TLGS)”, by Fatemeh Alizadeh et al.
However, some important issues need to be raised.

- Some drugs seem to have a beneficial effect on patients with chronic kidney disease. Please, briefly discuss this point by adding the reference (Irbesartan reduces the albumin excretion rate in microalbuminuric type 2 diabetic patients independently of hypertension: A randomized double-blind placebo-controlled crossover study. DOI 10.2337/diacare.25.11.19)

**Response: Agreed, we used this important study in the discussion section. (Line: 331-335)**

- The role of drugs as potential cofounders was not discussed. Please, briefly discuss this important issue.

**Response: Agreed, we discussed this important issue in the limitation part of the manuscript. (Line: 331-335)**

Reviewer #3: PONE-D-22-17476R1: statistical review

SUMMARY. This is a cohort study to evaluate the association between ideal cardiovascular health metrics (ICVHM) and time up an event of low estimated glomerular filtration rate (eGFR). The core statistical analysis relies on a battery of Cox regression models where the effect of ICVHM is adjusted by including available confounding factors. I have several concerns about the estimation method (major issue 1), sample selection (major issue 2), model specification (major issue 3). I also append some specific points that should be addressed.

MAJOR ISSUES.

1. Lines 178-180. Although data are interval censored, event times are estimated by mid-points and treated as they were exact times. This approach is a potential source of bias and it is not correct. The authors should repeat the statistical analysis by accounting for the additional uncertainty brought by interval censoring. Cox regression analysis for interval-censored data can be easily performed in Stata, which is the software exploited by the authors: see the command "stintcox".

**Response: The theory for the analysis of interval-censored data has been developed over the past four decades. The midpoint of the censoring interval is a common practice in analyzing interval-censored survival data in medical and reliability studies to simplify the interval censoring structure and then apply standard analytic techniques for multivariate survival data [1-4]. The availability of software for right censoring might well be the main reason for this simplifying practice [5]. Although the performance of the mid-time approach may lead to biased estimates because of limited information due to interval-censoring (the event might happen at the beginning or end of the follow-up duration) Cox analysis has minimal impact from the differing assumptions about the timing of events [6]. Furthermore, our research team just published several papers using a similar approach in high-impact journals, including the Plos One journal [7-9].**

**Despite this, as requested by the reviewer, to test the robustness of our findings, we performed Cox regression analysis with interval-censoring using the "stintcox" command in STATA as a sensitivity analysis. Accordingly, the results using the interval-censored approach did not differ from those that obtained from the mid-point approach. (Lines 204-206 and 252-254)**

2. Lines 91-97. The statistical analysis is made on a subset of the initial sample. To avoid issues of selection bias, the authors should explain why the final sample can be considered as a random subset of the initial sample. See also specific point 1 below.

**Response: Agreed, we more clarified the method section of the revised manuscript. Moreover, to address the selection bias [10], propensity score (PS), the estimated probability that a participant would have followed in the study, was computed using maximum likelihood logistic regression analysis among the study population. The multivariable analysis, including PS as another covariate, was performed as another sensitivity analysis; accordingly, the findings were essentially unchanged. So the issue of selection bias might not affect our data analysis. (Lines: 196-199 and 249-252)**

3. If I have understood well, covariates are included at baseline. However, some of this covariates (e.g. age) are time-varying and certainly have a time-varying confounding effects on the time up to the event. I'd welcome an analysis that adjusts for time-varying covariates. Alternately, the authors should explain why time-varying covariates are not appropriate here.

**Response: Agreed, to test the robustness of our findings, we treated covariates, including age and marital status, as time-varying confounders in the sensitivity analysis; our results remained essentially unchanged, as shown in the supplementary material. (Lines: 205-207 and 254-256)**

SPECIFIC POINTS
1. In lines 77-81, the authors talk about a large population-based study conducted in the Middle East and North Africa (MENA) during more than 15 years of follow-up. In lines 84-89, they instead talk about a 3-year study in Teheran, which seems the study considered in this paper. What is the link between the two studies? Why was the large population 15-year study evoked, if the actual study of interest is the 3-year study? Please clarify.

**Response: The TLGS study is a prospective cohort study started in 1999 and is planned to be continued for about 20 years for the non-communicable outcomes. The participant are follow up every 3 years (phase 1: 1999-2002, phase 2: 2002-2005, phase 3: 2005-2008, phase 4: 2008-2011, phase 5: 2011-2014, phase 6: 2014-2018, phase 7: 2018-2021) Detailed information regarding the TLGS is available elsewhere (trial registration number: ISRCTN52588395). The current study includes those who participated in phase 3 of TLGS (2005-2008) and followed till February 2021; the median follow-up time for the current study was 15 years. More description was added to the method section to clarify this issue [11, 12]. (Line: 96-97)**

2. Lines 182-184. I'm glad that the authors have made some diagnostics of the model. However, the results are declared but not displayed. These secondary results should be commented and displayed as supplementary material.

**Response: All P values for proportionality assumptions were > 0.1 in different multivariable-adjusted models. (Line: 193-194) and (Supplementary Tables S1 and S2)**

3. Data. It is good that all the data are available without restrictions in a repository. However, is this repository public? If it is not, then the authors should provide precise instructions for obtaining the data.

**Response: The TLGS data is not public, however, Azizi F. is the pioneer researcher of the TLGS, and other authors of this manuscript are among the research team of TLGS in the research institute for endocrine sciences (RIES), so we have asses to the TLGS data.**

4. There is a tendency to interpret the results as they were the outcome of a logistic regression model where the dependent variable is a binary variable that records the occurrence of the event. However, the dependent variable in a Cox model is the time up to the event and covariates modulate the hazard function. The text should be modified accordingly.

**Response: Agreed, we modified the discussion section accordingly. (Line: 259-267)**

5. There some typos across the text, please check.

**Response: Done.**

1.	Kim MY, Xue X. The analysis of multivariate interval‐censored survival data. Statistics in Medicine. 2002;21(23):3715-26.

2.	Rücker G, Messerer D. Remission duration: an example of interval‐censored observations. Statistics in Medicine. 1988;7(11):1139-45.

3.	Lin DY, Wei L-J. The robust inference for the Cox proportional hazards model. Journal of the American statistical Association. 1989;84(408):1074-8.

4.	Odell PM, Anderson KM, D'Agostino RB. Maximum likelihood estimation for interval-censored data using a Weibull-based accelerated failure time model. Biometrics. 1992:951-9.

5.	Gómez G, Calle ML, Oller R, Langohr K. Tutorial on methods for interval-censored data and their implementation in R. Statistical Modelling. 2009;9(4):259-97.

6.	Singh RS, Totawattage DP. The statistical analysis of interval-censored failure time data with applications. 2013.

7.	Derakhshan A, Sardarinia M, Khalili D, Momenan AA, Azizi F, Hadaegh F. Sex specific incidence rates of type 2 diabetes and its risk factors over 9 years of follow-up: Tehran Lipid and Glucose Study. PloS one. 2014;9(7):e102563.

8.	Ramezankhani A, Azizi F, Hadaegh F. Associations of marital status with diabetes, hypertension, cardiovascular disease and all-cause mortality: A long term follow-up study. PloS one. 2019;14(4):e0215593.

9.	Derakhshan A, Bagherzadeh‐Khiabani F, Arshi B, Ramezankhani A, Azizi F, Hadaegh F. Different combinations of glucose tolerance and blood pressure status and incident diabetes, hypertension, and chronic kidney disease. Journal of the American Heart Association. 2016;5(8):e003917.

10.	Rubin DB. Estimating causal effects from large data sets using propensity scores. Annals of internal medicine. 1997;127(8_Part_2):757-63.

11.	Azizi F, Rahmani M, Emami H, Mirmiran P, Hajipour R, Madjid M, et al. Cardiovascular risk factors in an Iranian urban population: Tehran lipid and glucose study (phase 1). Sozial-und präventivmedizin. 2002;47(6):408-26. doi: 10.1007/s000380200008.

12.	Azizi F, Ghanbarian A, Momenan AA, Hadaegh F, Mirmiran P, Hedayati M, et al. Prevention of non-communicable disease in a population in nutrition transition: Tehran Lipid and Glucose Study phase II. Trials. 2009;10(1):1-15. doi: 10.1186/1745-6215-10-5.
